# Untargeted Metabolomic Approach of *Curcuma longa* to Neurodegenerative Phytocarrier System Based on Silver Nanoparticles

**DOI:** 10.3390/antiox11112261

**Published:** 2022-11-16

**Authors:** Adina-Elena Segneanu, Gabriela Vlase, Alexandra Teodora Lukinich-Gruia, Dumitru-Daniel Herea, Ioan Grozescu

**Affiliations:** 1 Institute for Advanced Environmental Research, West University of Timisoara (ICAM-WUT), Oituz nr. 4, 300086 Timisoara, Romania; 2 Res. Ctr. Thermal Anal Environm Problems, West University of Timisoara, Pestalozzi St. 16, 300115 Timisoara, Romania; 3 OncoGen Centre, Clinical County Hospital “Pius Branzeu”, Blvd. Liviu Rebreanu 156, 300723 Timisoara, Romania; 4 National Institute of Research and Development for Technical Physics, 47 Mangeron Blvd, 700050 Iasi, Romania; 5 CAICON Department, University Politehnica Timisoara, 2 P-ta Victoriei, 300006 Timisoara, Romania

**Keywords:** secondary metabolites, curcuma, mass spectra, silver nanoparticles, phytocarrier system, antioxidant activity

## Abstract

Curcuma is one of the most famous medicinal and tropical aromatic plants. Its health benefits have been appreciated and exploited in traditional Asian medicine since ancient times. Various studies have investigated its complex chemical composition and demonstrated the remarkable therapeutic properties of curcuma’s phytoconstituents. Oxidative stress is a decisive driving factor triggering numerous pathologies (neurodegenerative, psychiatric and cardiovascular diseases; diabetes; tumors, etc.). Numerous recent studies have focused on the use of natural compounds and nanomaterials as innovative molecular targeting agents as effective therapeutic strategies. In this study, we report, for the first time, the development of a simple target phytocarrier system that capitalizes on the bioactive properties of curcuma and AgNPs. The complete metabolic profile of curcuma was determined based on gas chromatography-mass spectrometry (GC-MS) and electrospray ionization quadrupole time-of-flight mass spectrometry (ESI-QTOF-MS). A total of 80 metabolites were identified under mass spectra (MS)-positive mode from 10 secondary metabolite categories: terpenoids, amino acids, diarylheptanoids, flavonoids, phenolic acids, steroids, fatty acids, coumarins, alkaloids and miscellaneous. In addition, the biological activity of each class of metabolites was discussed. A comprehensive characterization (FT-IR, UV-Vis, DLS, SEM, TEM, EDS, zeta potential and XRD) was performed to study the morphostructural properties of this new phytocarrier system. Antioxidant activity of the new phytocarrier system was evaluated using a combination of in vitro methods (total phenolic assay, 2,2-Diphenyl-1-picrylhydrazyl (DPPH) radical scavenging assay and cyclic voltammetric method (Trolox equivalent antioxidant capacity (TEAC) electrochemical assay)). Antioxidants assays showed that the phytocarrier system exhibits superior antioxidant properties to those of its components, i.e., curcuma or citrate-coated-AgNPs. These data confirm the potential to enhance relevant theoretical knowledge in the area of innovative antioxidant agents, with potential application in neurodegenerative therapeutic strategies.

## 1. Introduction

*Curcuma longa* L., a plant that belongs to the *Zingiberaceae* family, has played an important role in Indian culture since ancient times. The well-known turmeric spice is obtained from the rhizome of this plant, the applications of which include multiple culinary preparations, dyes, cosmetics, religious rituals and traditional medicine [1].

In traditional Asian medicine, turmeric (curcuma) is used as an anti-inflammatory, antiseptic, analgesic, antibacterial, digestive, carminative and cholagogue, as well as to treat anorexia and respiratory disorders (cough, sinusitis and asthma) [1,2]. Recent research reported that curcumin, one of the biologically active compounds in turmeric, has multiple therapeutic properties, such as anti-inflammatory, antidiabetic, antiviral, antioxidation, antitumoral, neuroprotective, angiogenic, antiatherosclerosis and hepatoprotective activity [1,2,3,4,5].

Curcuma’s considerable pharmacological activity is the result of the joint and synergistic action of its numerous phytoconstituents (curcuminoids, volatile oils, terpenoids, sterols, fatty acids, phenolic acids and flavonoids) [1,6,7]. Various studies paid a special attention the isolation and synthesis of curcumin, investigating its biological activity [3,4,8]. Nevertheless, the use of curcumin in biomedical and food applications is limited, given that it is a polyphenol and hydrophobic, with low chemical stability, especially in acidic pH, which is why it has low bioavailability [9,10].

Owing to their size, many phytoconstituents have limited adsorption capacity and implicitly low bioavailability, which represents one of the main issues associated with drug discovery based on natural compounds. Another concern is attributed to the variation in metabolic composition depending on various biotic and abiotic parameters, as well as the highly complex chemical structure with numerous chiral centers [11,12]. However, the current trend in traditional medicine offers the advantage of using natural products, eliminating the multiple side effects of synthetic drugs. Consequently, recent studies have focused on the design of innovative materials based on natural products, with increased pharmacological efficiency, controlled release, specific site targeting and extended action [11,12]. In this sense, special attention had been paid to phytoconstituents with neuroprotective, antitumor, antioxidant and anti-inflammatory activity [11].

It is well known that nanoparticles have particular physicochemical and biological properties. Therefore, various types of nanoparticles have been investigated for biomedical applications, such as silver, gold, iron oxide and silica. However, gold and silver nanoparticles stand out, proving superior owing to their high biocompatibility and versatility [11,12]. Medical applications of silver have been known since ancient times [13]. For a long time, silver was used in the manufacture of commonly used objects, the main purpose of which was to limit the spread of diseases [13,14]. In the Middle Ages, argyria, a skin condition resulting from overexposed to silver, was associated with the high society. Until the early 20th century, silver was used extensively for its disinfectant properties. The development and use of antibiotics have led to a drastic decline in the biomedical applications of silver. However, this generated a secondary phenomenon in short order; the resistance of bacteria to antibiotics was a turning point that demanded the identification of more effective therapeutic strategies [14,15,16,17,18].

The implementation of nanotechnology in medicine has contributed to not only overcoming these obstacles but also the development of nanomaterials and nanodrug delivery systems with outstanding performance [12]. Hence, the latest nanodelivery systems are the result of the association of natural products with nanoparticles (by encapsulating or attaching biomolecules) with very high biological activity and minimal toxicity. Studies have reported that these nanostructures can mitigate the effects of drug resistance, opening up new therapeutic possibilities [11,12,19,20].

Several methods have recently been developed to improve the physicochemical properties of curcumin, including encapsulation or the use of different carriers [4]. The association of curcumin with different metal nanoparticles can produce efficient therapeutic agents (antioxidant, antitumoral and neuroprotective). The physicochemical properties of curcumin are modified in these complexes (increase in aqueous solubility, stability at physiological pH and bioavailability), in addition to improving metal dissolution and minimizing metal toxicity [4,8,19,21,22]. Various studies have investigated the synthesis of curcumin complexation with different metallic nanoparticles, such as magnetic, silver, copper and manganese nanoparticles, in order to increse its absorption in the body and metabolic stability [8,22,23,24].

Moreover, recent research has reported that silver nanoparticles (AgNPs) have high microbial, antifungal, antiviral and antitumoral activity. AgNPs have the ability to cross various biological barriers in the human body [14,15,16,17,23,25].

The biological mechanism of AgNPs is not fully understood. The antimicrobial activity of silver nanoparticles has been suggested to be the result of membrane interaction and microbial DNA damage [23,26,27]. Owing to their remarkable physicochemical and biological properties, silver nanoparticles currently have multiple applications in diverse fields, including medicine (detection, diagnosis, therapy, drug delivery, antimicrobial agents, wound treatment, dressings, vaccines, biomaterials for orthopedics and dentistry, disinfectants, etc.), biosensors, the food industry, renewable energy (cell fuels, solar cells, etc.), cosmetics (sunscreen, deodorants, toothpaste, etc.), textiles, advanced materials (electronics, ceramics, catalysts, dyes, etc.), drinking water treatment, etc. [27,28,29].

Owing to their low reactivity, spherical AgNPs are one of the ingredients of the COVID-19 vaccine [15,16]. Although the toxicity of silver is the subject of many controversies concerning biomedical applications, recent studies have shown that the biological activity of silver is correlated with size, surface area, particle shape and dosage. It has been shown that the smaller the silver nanoparticles, the more harmless to the human body [14,15,16,23,26,27,30]. Coating materials represent another method to reduce the toxicity of AgNPs [17,26,31,32]. Several recent studies have investigated methods of obtaining different types of curcumin complexes and silver nanoparticles (coencapsulation, nanoformulation, hydrogels, one-pot synthesis, etc.) for various biomedical applications [19,22,23,24,26].

The latest research reported the use of antioxidant agents as an innovative and efficient approach to the treatment of neurodegenerative diseases [33,34,35,36,37,38].

Phytoconstituents such as phenolic compounds, vitamin C, flavonoids, vitamin E, sulphated polysaccharides, carotenoids, sterols, etc., have exhibit high antioxidant activity, making them ideal candidates for neuroprotective therapy. However, studies have shown that the in vivo bioavailability of these phytoconstituents is poor, with low permeability across the blood–brain barrier [26,27,35,39,40,41,42,43].

Studies have reported that curcumin exerts antioxidant and neuroprotective activity. Moreover, curcumin intercedes in the neuroprotective mechanism associated with neurodegeneration by inhibiting the inflammatory process and minimizing oxidative stress [40,42,44]. Therefore, it is necessary to develop strategies to enhance the biological activity and thus the metabolic dissolution of these natural antioxidants. A drug delivery system is one of the most effective methods to ensure the controlled release of antioxidant agents directly to a specific site [36,37,42].

Substrate antioxidant activity is the result of the presence of a heterogeneous category of bioactive compounds, including polyphenolics, flavonoids, phenolic acids, phytate carotenoids, vitamin C, vitamin E, vitamin K, coumarins, stilbenes, resveratrol, lipoic acid, curcumin, pyridinols, pyrimidinols, thiols, disulfides, hydrocarbons, lactones, non-phenolic terpenoid (limonene, linalool, citral and gamma-terpinene), trisubstituted aromatic amines and transition metal ions (Ag, Au and Zn) [45,46,47,48,49,50,51].

Plants contains a heterogeneous mixture of phytoconstituents with biological and synergistic activity, which is why it is necessary to evaluate their antioxidant activity through several tests that address the various mechanisms of action of a particular category of components [52]. Moreover, the antioxidant activity of a plant depends on various factors, including variety, morphostructural composition, abiotic factors (climate and soil parameters) and harvest period [52].

Antioxidants can be categorized according to activity, size, solubility, origin and other factors. There are two categories of antioxidants in terms of mode of actions: direct antioxidants (prevent, inhibit or minimize the oxidation process) and indirect antioxidants (determine the increase in antioxidant capacity through antioxidant enzyme expression) [45,53].

Direct antioxidants are divided into preventive antioxidants (metal-chelating agents: phytate or curcumin), chain-breaking antioxidants or radical-trapping antioxidants (polyphenolics, phenolic acids and ascorbic acid) and termination-enhancing antioxidants (non-phenolic terpenoid) [45,53]. Their main characteristics are redox activity, as well as the ability to be consumed or undergo chemical changes and be replenished or regenerated.

Antioxidant performance depends mainly on rate constant and thermodinamic parameters of reaction with a specific free radical, as well as a series of physicochemical factors (chemical structure, type of substrate oxidation position, concentration and temperature), the presence of other pro-oxidants or synergists, bioavailability and metabolism pathway [45,54].

The development of a nanocarrier depends on the morphostructural parameters of the nanoparticles (size, shape, surface charge, etc.) and the biological activity of its components [12].

Silver nanoparticles, owing to their unique size and versatility, high surface-to-volume ratio, biocompatibility and mobility, are able to cross the blood–brain barrier (BBB), which represent a major limitation in the current development of active drugs [12,13,14,15,16,20,27,29]. The design of a performant neuroprotective phytoengineered carrier system based on AgNPs represents an innovative alternative that can overcome the limitation of current therapeutic strategies, tracing specific organ and molecular targets linked to neurodegeneration.

To the best of our knowledge, this study is the first to investigate the development of a target-specific nanocarrier delivery system that capitalizes on the therapeutic properties of bot curcuma and AgNPs. We evaluated the morphostructural and antioxidant properties of the proposed phytocarrier system.

## 2. Materials and Methods

All reagents used in this study were of analytical grade. Methanol, chloroform, dichloromethane and ethanol were purchased from Sigma Aldrich (München, Germany) and used without further purification. DPPH (2,2-diphenyl-1-picrylhydrazyl), β-carotene type II, synthetic (≥95%), ascorbic acid, AgNO_3_, sodium citrate, sodium carbonate, Folin–Ciocalteu phenol reagent (2N), 2,2′-azino-bis(3-ethylbenzothiazoline-6-sulphonic acid) diammonium salt (ABTS salt), 6-hydroxy-2,5,7,8-tetramethychroman-2-carboxylic acid (Trolox), potassium persulfate and potassium chloride of 99% purity or higher were purchased from Sigma Aldrich (München, Germany). Curcumin and propyl gallate (purum) were purchased from Fluka (Buchs, Switzerland). *Curcuma* (*Curcuma longa* L.) root powder was purchased from a local market in Dortmund, Germany. Double distilled water (DDW) was used throughout the experiments.

### 2.1. Preparation of Phytocarrier System Components 

AgNPs synthesis: Citrate synthesis of silver nanoparticles was carried out using the following procedure. An amount of 0.64 g AgNO_3_ was added to 1000 mL ultrapure water, followed by the addition of 50 mL of sodium citrate solution (1.6%). The mixture was kept at 90 °C for 90 min and stirred at 800 rpm. Then, 0.8 g ascorbic acid was added, and the obtained suspension was stirred at 90 °C for another 40 min.

### 2.2. Plant Preparation for Chemical Screening

For each analysis, 2.0 g of curcuma powder was subjected to sonication extraction in 35 mL of solvent (methanol/chloroform = 1:1) for 35 min at 37 °C with a frequency of 50 kHz. The solution was concentrated using a rotavapor, and the residue was dissolved in MeOH. The extract was centrifuged, and the supernatant was filtered through a 0.2 µm syringe filter and stored at −18 °C until MS analysis. All samples were prepared in triplicate.

### 2.3. GC-MS Analysis

Gas chromatography was carried on an HP6890 gas chromatograph coupled with an HP5973 mass spectrometer (Agilent Technologies, Santa Clara, CA, USA) with a Bruker Br-5MS capillary column (30 m × 0.25 mm id × 0.25 μm) (Bruker, Fremont, CA, USA) under helium with a flow of 1 mL/min.

### 2.4. GC-MS Separation Conditions

The oven temperature was increased from 50 °C to 300 °C at a rate of 6 °C/min and held at this temperature for 5 min. The temperature of the injector was 280 °C, and the temperature at the interface was 220 °C. The mass of the compounds was registered at 70 eV ionization energy starting after 3 min of solvent delay. The mass spectrometer was source-heated at 230 °C, and the MS Quad was heated at 150 °C. The mass values of the identified compounds were scanned from 50 to 550 amu. Compounds were identified based on their mass spectra, which were compared to the NIST0.2 mass spectra library database (USA National Institute of Science and Technology software, (NIST, Gaithersburg, MD, USA). Retention indices (RIs) were also calculated for each compound based on a C_8_–C_20_ alkane standard mixture calibration curve, which was compared to Adams indices in the literature [55].

A percentage area of each compound was calculated by summing all the experimental integrated peak areas (Table 1).

### 2.5. Mass Spectrometry

MS experiments were conducted on a Bruker EIS-QTOF-MS (Daltonics, Bremen, Germany). The full mass spectra were acquired in positive ion mode at the temperature source block temperature (80 °C) and at 25–85 eV collision energy. The scan range of full MS was m/z 100–3000, with a scan speed of 2.0 scans/s. Peak assignment to specific metabolites was based on the NIST/NBS-3 standard library spectral database (National Institute of Standards and Technology/National Bureau of Standards) (NIST, Gaithersburg, MD, USA). The mass spectra of the identified compounds are presented in Table 2.

### 2.6. Phytocarrier System Preparation

For each analysis, 2.0 g of sample was prepared from curcuma powder, and a AgNPs solution was added (curcuma/AuNPs nanoparticles = 1:3) at room temperature (23 °C) and magnetically stirred (450 rpm) for 22 h. The obtained mixture was filtered (F185 mm filter paper) and dried in an oven at 35 °C for 5 h. For TEM analysis, a 0.22 nm filter was used to filter the curcuma powder.

### 2.7. Characterisation of the Phytocarrier System

UV–Vis Analysis: The UV-Vis spectra were obtained using a T90+ UV-Vis spectrophotometer (PG Instruments, UK) with a double beam in the photometric range of 190–900 nm. All absorbance measurements were taken in a 10 mm UV/Vis spectroscopy cell at room temperature, using ethanol as a blank.

### 2.8. Fourier Transform Infrared (FTIR) Spectroscopy

Data collection was performed after 20 recordings at a resolution of 4 cm^−1^ in the range of 4000–400 cm^−1^ on a Shimadzu AIM-9000 with ATR devices.

### 2.9. XDR Spectroscoy

X-ray powder diffraction (XRD) was performed using a Rigaku Ultima IV diffractometer equipped with a D/teX ultra-detector operating at 40 kV and 40 mA with monochromatic CuKα radiation (λ = 1.5406 Å) in the 2θ range of 10–80°, with a scan speed of 5°/min and a step size of 0.01°. The XRD patterns were compared with those from the ICDD Powder Diffraction Database (ICDD file 04-015-9120). The average crystallite size and the phase content were calculated using the whole-pattern profile-fitting method (WPPF).

### 2.10. Scanning Electron Microscopy (SEM)

SEM micrographs were obtained with an SEM-EDS system (QUANTA INSPECT F50) equipped with a field-emission gun (FEG) with a 1.2 nm resolution and an energy-dispersive X-ray spectrometer (EDS) with an MnK resolution of 133 eV.

### 2.11. Dynamic Light Scattering (DLS) Particle Size Distribution Analysis

DLS analysis was perfomed on a Microtrac/Nanotrac 252 (Montgomeryville, PA, USA). Each sample was analyzed in triplicate at room temperature (23 °C) at a scattering angle of 172°.

### 2.12. High-Resolution Transmission Electron Microscopy (HR-TEM)

The morphology of silver particles and phytocarriers were determined through an ultra-high resolution transmission electron microscope (UHR-TEM) (Libra 200 UHR-TEM, Carl Zeiss, Germany).

### 2.13. Zeta Potential Analysis

The zeta potential was determined on an AMERIGO particle size and zeta potential analyzer (Pessac, France), with six measurements/s, and electrode distance of 5 mm, a temperature of 25 °C, conductivity of 5.10 V, carrier frequency of 8210 Hz, reference intensity of 2660 kcps, applied field of 20.27 V/cm and scattering intensity of 2850 kcps.

### 2.14. Antioxidant Activity

The antioxidant activity of the phytocarrier system was evaluated using three assays: a 2,2-diphenyl-1-picrylhydrazyl (DPPH) radical scavenging assay, a Folin–Ciocalteu assay and cyclic voltammetry (Trolox Equivalent Antioxidant Capacity (TEAC) electrochemical assay).

The phytocarrier system (0.3 g) and curcuma (0.5 g) samples were dissolved in methanol (9 mL and 15 mL, respectively). The mixtures were stirred at room temperature (23 °C) for 24 h, then centrifuged at 3000 rpm for 5 min. The supernatant was then collected for use in the antioxidant assays (2,2-diphenyl-1-picrylhydrazyl (DPPH) radical scavenging assay, Folin–Ciocalteu assay (cyclic voltammetry method). The AgNP solution was used without further preparation for the cyclic voltammetry method.

### 2.15. Determination of Total Phenolic Content

The total phenolic content in the phytocarrier system and curcuma samples was determined according to the Folin–Ciocalteu method adapted from the literature [56].

A volume of 0.1 mL of each sample was used for total phenolic content assays. Folin–Ciocalteu reagent 2 N was diluted 1:10 in distilled water. A ratio of sample/Folin–Ciocalteu reagent 0.2 N (1:5) was prepared, vortexed and kept at room temperature (23 °C) for 5 min in the dark. The reaction between samples and Folin–Ciocalteu reagent was allowed to complete, before adding an equal volume of Folin–Ciocalteu reagent and 7.5% sodium carbonate (Na_2_CO_3_) solution. The obtained mixture was vortexed and kept at room temperature (23 °C) for 1 h in the dark. The samples were scanned by a UV-Vis spectrophotometer (Tecan i-control, 1.10.4.0 infinite 200Pro spectrophotometer with Corning 96 Flat Bottom clear Polystyrol plates, Tecan, Männedorf, Switzerland) for 10 to 40 min after the addition of the sodium carbonate solution and scanned from 400 to 900 nm with intervals of 2 min between each reading to determine the spectra. The absorbance was measured at 725 nm.

The results are expressed in gallic acid equivalents (mg GAE/g sample) using a propyl gallate standard calibration curve between 1 mg/mL and 12.5 µg/mL in methanol.

Sample extract concentrations were calculated based on the linear equation obtained from the standard curve (y = 0.8353x − 0.1207) [57].

### 2.16. DPPH Radical Scavenging Assay

The antioxidant activities of the phytocarrier system, curcuma sample and three antioxidant standards (ascorbic acid, curcumin and beta-carotene) were comparatively evaluated by a DPPH (2,2-diphenyl-1-picrylhydrazyl) free radical elimination assay.

A stock solution of 1 mg/mL DPPH and dilutions for a calibration curve with a range of concentrations between 3.12 µg/mL and 0.1 mg/mL were prepared, with the following serial dilutions of standards: ascorbic acid and curcumin were combined with a stock solution of 1 mg/mL, and between 0.1 mg/mL and 0.2 µg/mL was diluted in methanol; β-carotene started from 1.66 mg/mL, and between 0.83 mg/mL and 41.66 µg/mL was diluted in a solvent ratio of 2:1 methanol/dichloromethane.

Sample extracts of and the phytocarrier system had dilutions between 0.33 mg/mL and 2.08 µg/mL for curcuma and between 0.33 mg/mL and 4.16 µg/mL for the phytocarrier system. The ratio (*v*/*v*) of DPPH to samples was of 1:1.

All solutions were prepared in four replicates in methanol, and 1:1 DPPH 0.1 mg/mL and methanol was used as a control sample.

For β-carotene, methanol/dichloromethane (2:1) was used as a control solvent.

All samples were pipetted on 96-well plates and incubated in the dark at room temperature for 30 min. After the reaction was stabilized, the absorbance was measured, and readings were performed at 515 nm. The obtained results were used to calculate the average and the inhibition percentage (Inh%). Inh% was further used to obtain the IC_50_ value, which was calculated based on the equation of a calibration curve generated for each sample and standard, with results presented as calculated Inh% versus concentration (µg/mL).

### 2.17. Statistical Analysis

All results were obtained with Microsoft Office Excel 2010. Data were subjected to the average of four replicates for all samples, all calibration curves and concentrations.

### 2.18. Trolox Equivalent Antioxidant Capacity (TEAC) Electrochemical Assays

The stock solutions of ABTS and Trolox were prepared by dissolution in methanol. Further dilutions were achieved with Millipore ultrapure water (18 MΩ∙cm).

Voltammetric measurements (cyclic voltammetry, CV) were performed on an Autolab PGSTAT101 apparatus (Eco Chemie BV, Utrecht, The Netherlands) using NOVA 2.2 software in a standard three-electrode electrochemical cell with a carbon paste working electrode (Metrohm, 3 mm in diameter), a platinum wire (CH Instruments) as the counter electrode and an Ag/AgCl reference electrode saturated with 3 M KCl (Methrom, Switzerland). All experiments were run at room temperature.

The TEAC electrochemical assays were performed according to the method reported in the literature [58].

A solution of the stable radical cation ABTS∙^+^ was prepared by mixing 7 mM ABTS with 2.45 mM persulfate in a volumetric ratio of 1:1 in methanol. The mixture was kept in the dark for 8 h at 20 °C prior use to ensure the quantitative conversion of ABTS to ABTS∙^+^. The stock solution of ABTS∙^+^ was suitably diluted with various amounts of methanol to achieve significant voltammetric outputs during the CV measurements. All working solutions of ABTS∙^+^ contained 0.05 M KCl as the supporting electrolyte.

A volume of 250 μL of each sample (phytocarrier system, curcuma and citrate-coated AgNPs) was added to the working solutions of ABTS∙^+^, and the reaction mixtures were left to react for 2–3 min. Several CVs were recorded to evaluate the stability of the reaction mixture and the reproducibility of the datasets. The reaction between ABTS∙^+^ and Trolox was used to build the calibration plot. Several aliquots of 1 mM Trolox solution were mixed with ABTS stock solution to yield a final ABTS concentration of 0.12 mM and a Trolox concentration range of 0.02–0.12 mM. For each sample, at least 10 consecutive voltammograms were recorded, and the average response was recorded. The residual standard deviations (RSD) in all assays were below 0.25%. The peak currents were processed with Nova 2.2 software and referenced to the baseline.

## 3. Results and Discussions

Plants are an invaluable resource for biomolecules with high therapeutic potential. A considerable variety of secondary metabolites with complex chemical structures has been identified in plants [59,60].

Studies have shown that the stress conditions dictated by the environment in which a particular plant develops influence the production of secondary metabolism and the structural and functional relationships between them [61,62,63,64,65,66].

Moreover, the proportion of volatile compounds and curcuminoids, considered the main bioactive compounds in curcuma, is also influenced by experimental conditions (extraction, etc.) [67].

Accordingly, the biological activity of a specific plant is dictated by the composition and the synergistic and complementary interaction of their secondary metabolites. The combined action of all metabolites in the plant leads to increased biological activity and metabolic stability of an active component [68,69]. Therefore, it is very challenging to establish the interdependence between the therapeutic effect and the chemical composition of a plant. In some cases, in vivo studies do not support the mechanism of action of a particular secondary metabolite isolated from a plant [10].

Given the remarkable economic value of curcuma, studies on turmeric continue to be relevant, especially with respect to its therapeutic potential.

Secondary metabolite screening of curcuma was tentatively carried out via gas-chromatography coupled with mass spectroscopy (GC-MS) and electrospray ionization–quadrupole time-of-flight mass spectrometry (ESI-QTOF-MS) analysis.

**Figure 1:**
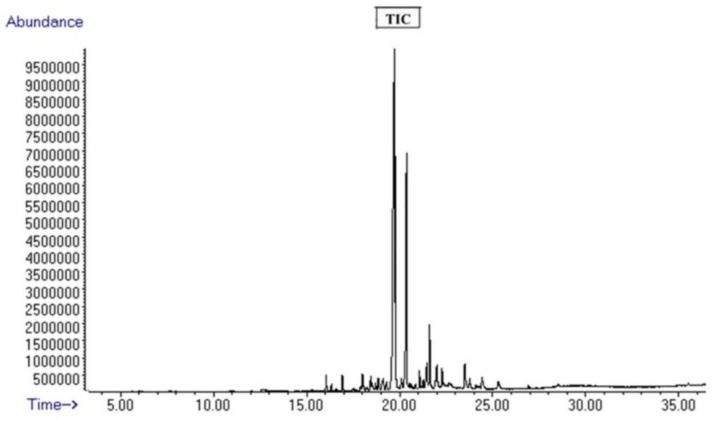
TIC chromatogram of curcuma extract.

The GC-MS analysis (Figure 1) revealed the separation of some low-molecular-weight metabolites from the curcuma sample.

The results are summarized in Table 1, including the identification of tentative GC-MS compounds from the curcuma sample.

**Table 1:** Main compounds identified by GC-MS analysis of a curcuma sample.

No	Retention Time (RT)	Retention Index (RI)	Adams Index (AI)	Area%	Compound Name	Ref.
1	14.771	1439	1417	0.36	α-caryophyllene	[70]
2	16.057	1502	1479	8.84	Ar-curcumene	[71,72,73,74,75]
3	16.337	1516	1493	0.71	α-zingiberene	[74,75,76,77,78]
4	16.6	1529	1505	1.14	β-cisabolene	[70,74,75,76]
5	16.92	1545	1521	1.08	β-sesquiphellandrene	[70,72,74,75,76]
6	18.515	1624	1718	0.42	santalol, cis,β-	[75,76]
6	19.618	1678	1668	50.01	Ar-turmerone	[72,74,75]
7	19.703	1682	1664	8.70	turmerone	[72,74,75,76,77]
8	20.315	1713	1699	18.47	curlone	[70,72,75,76,77,78]

RI—retention index calculated based on a calibration curve of a C8–C20 alkane standard mixture.

The GC-MS analysis revealed the presence of eight major components, accounting for 89.7% of the total peak area in curcuma samples (Figure 1).

### 3.1. Mass Spectrometric Analysis of Curcuma Sample

The spectra revealed a complex mixture of low-molecular-weight metabolites molecules, among which only a few were detected. The mass spectra of identified components were compared with those of the NIST/EPA/NIH Mass Spectral Library 3.0 database, in addition to a literature review [67,79,80,81,82].

**Figure 2:**
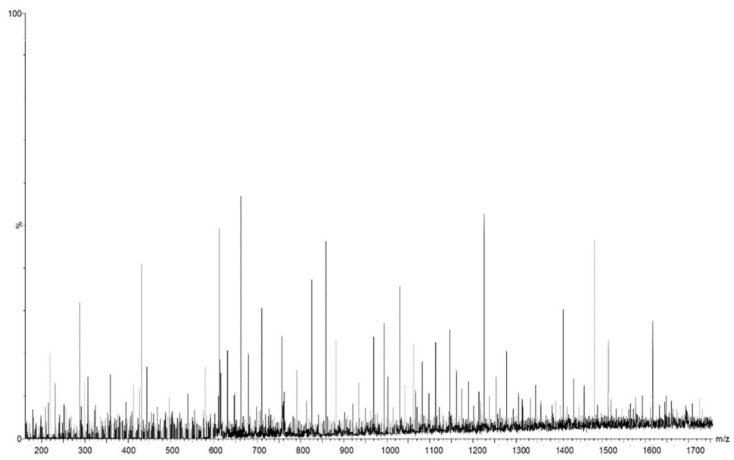
The mass spectrum of *Curcuma longa*.

The mass spectrum and the phytoconstituents identified by ESI-QTOF-MS analysis are presented in Figure 2 and Table 2, respectively.

**Table 2:** The metabolites identified through electrospray ionization-quadrupole time-of-flight mass spectrometry (ESI-QTOF-MS) analysis.

No	Detected m/z	Theoretic m/z	Formula	Tentative Identification	Category	Ref.
1	75.09	75.07	C_2_H_5_NO_2_	glycine	amino acid	[83]
2	89.08	89.09	C_3_H_7_NO_2_	alanine	amino acid	[83]
3	105.08	105.09	C_3_H_7_NO_3_	serine	amino acid	[83]
4	115.11	115.13	C_5_H_9_NO_2_	proline	amino acid	[83]
5	117.14	117.15	C_5_H_11_NO_2_	valine	amino acid	[83]
6	120.19	120.19	C_9_H_12_	trimethylbenzene	miscellaneous	[84]
7	131.15	131.17	C_6_H_13_NO_2_	leucine	amino acid	[73]
8	133.11	133.10	C_4_H_7_NO_4_	aspartic acid	amino acid	[83]
9	134.21	134.22	C_10_H_14_	cymene	terpenoid	[85]
10	136.22	136.23	C_10_H_16_	limonene	terpenoid	[86]
11	136.24	136.23	C_10_H_16_	terpinene	terpenoid	[65,85]
12	146.12	146.14	C_9_H_6_O_2_	coumarin	coumarin	[82]
13	147.11	147.13	C_5_H_9_NO_4_	glutamic acid	amino acid	[83]
14	148.15	148.16	C_9_H_8_O_2_	cinnamic acid	phenolic acid	[87]
15	149.22	149.21	C_5_H_11_NO_2_S	methionine	amino acid	[83]
16	150.23	150.22	C_10_H_14_O	tymol	terpenoid	[75,77]
17	152.24	152.23	C_10_H_16_O	thujone	terpenoids	[88]
18	154.11	154.12	C_7_H_6_O_4_	protocatechuic acid	phenolic acid	[82]
19	154.27	154.25	C_10_H_18_O	terpineol	terpenoid	[85]
20	164.17	164.16	C_9_H_8_O_3_	p-coumaric acid	phenolic acid	[86]
21	164.19	164.20	C_10_H_12_O_2_	eugenol	terpenoids	[86]
22	165.21	165.19	C_9_H_11_NO_2_	phenylalanine	amino acid	[83]
23	170.13	170.12	C_7_H_6_O_5_	gallic acid	phenolic acid	[82]
24	170.30	170.29	C_11_H_22_O	undecan-2-one	miscellaneous	[74]
25	174.21	174.20	C_6_H_14_N_4_O_2_	arginine	amino acid	[83]
26	180.15	180.16	C_9_H_8_O_4_	caffeic acid	phenolic acid	[86]
27	182.29	182.30	C_12_H_22_O	dodec-11-en-2-one	miscellaneous	[84]
28	192.23	192.21	C_11_H_12_O_3_	dehydrozingerone	flavonoids	[84,89]
29	194.19	194.18	C_10_H_10_O_4_	ferulic acid	phenolic acid	[82]
30	194.24	194.23	C_11_H_14_O_3_	zingiberone	terpenoids	[79]
31	202.31	202.33	C_15_H_22_	β-curcumene	terpenoid	[85]
32	204.33	204.35	C_15_H_24_	zingiberene	terpenoid	[85]
33	216.33	216.32	C_15_H_20_O	turmerone	terpenoid	[85]
34	218.31	218.33	C_15_H_22_O	germacrone	terpenoid	[85,89]
35	220.33	220.35	C_15_H_24_O	turmerol	terpenoid	[85,89]
36	204.34	204.35	C_15_H_24_	zingibrene	terpenoids	[74,75,87,88,89,90]
37	216.31	216.32	C_15_H_20_O	curzerene	terpenoid	[89,91]
38	218.34	218.33	C_15_H_22_O	a-costal	terpenoid	[75]
39	220.36	220.35	C_15_H_24_O	santanol	terpenoid	[74]
40	222.34	222.35	C_15_H_24_O	a-copaen-11-ol	terpenoid	[75]
41	222.26	222.37	C_15_H_26_O	a-bisabolol	terpenoid	[87,88]
42	230.29	230.30	C_15_H_18_O_2_	curzerenone	terpenoid	[74,85]
43	232.33	232.32	C_15_H_20_O_2_	glechomanolide	terpenoids	[89]
44	234.31	234.33	C_15_H_22_O_2_	curcumenol	terpenoids	[83]
45	236.37	236.35	C_15_H_24_O_2_	curdione	terpenoids	[89]
46	246.31	246.30	C_15_H_18_O_3_	zederone	terpenoids	[89]
47	250.32	250.33	C_15_H_22_O_3_	procurcumadiol	terpenoids	[86]
48	252.33	252.35	C_15_H_24_O_3_	bisacurone	terpenoids	[92]
49	254.23	254.24	C_15_H_10_O_4_	2-hydroxy-3-(hydroxymethyl)anthraquinone	miscellaneous	[89]
50	256.41	256.42	C_16_H_32_O_2_	palmitic acid	fatty acid	[67]
51	270.23	270.24	C_15_H_10_O_5_	apigenin	flavonoids	[83]
52	272.23	272.25	C_15_H_12_O_5_	naringenin	flavonoids	[80]
53	280.39	280.40	C_18_H_32_O_2_	linoleic acid	fatty acid	[67]
54	282.49	282.5	C_18_H_34_O_2_	oleic acid	fatty acid	[67]
55	284.51	284.5	C_18_H_36_O_2_	stearic acid	fatty acid	[67]
56	290.28	290.27	C_15_H_14_O_6_	epicatechin	flavonoids	[82]
57	290.39	290.4	C_19_H_30_O_2_	methyl stearidonate	fatty acids	[84]
58	302.22	302.23	C_15_H_10_O_7_	quercetin	flavonoids	[80]
59	308.29	308.30	C_19_H_16_O_4_	bisdemethoxycurcumin	flavonoids	[85]
60	316.39	316.4	C_19_H_24_O_4_	1,7-bis(4-hydroxyphenyl)-3,5-heptanediol	diarylheptanoid	[93]
61	318.21	318.23	C_15_H_10_O_8_	myricetin	flavonoids	[82]
62	326.29	326.3	C_19_H_18_O_5_	1,5-bis(4-hydroxy-3-methoxyphenyl)-1,4-pentadien-3-one	diarylheptanoid	[93]
63	354.29	354.31	C_16_H_18_O_9_	chlorogenic acid	phenolic acid	[82]
64	368.37	368.39	C_21_H_20_O_6_	curcumin	diarylheptanoid	[85]
65	368.41	368.40	C_21_H_20_O_6_	cyclocurcumin	diarylheptanoid	[92]
66	370.39	370.4	C_21_H_22_O_6_	1,2-dihydrocurcumin	diarylheptanoid	[93]
67	390.49	390.5	C_23_H_34_O_5_	gitoxigenin	steroids	[93]
68	386.39	396.40	C_23_H_24_O_6_	dimethoxycurcumin	diarylheptanoid	[85]
69	386.69	386.70	C_27_H_46_O	cholesterol	sterols	[86]
70	400.68	400.70	C_28_H_48_O	campesterol	sterols	[86]
71	402.49	402.50	C_25_H_26_N_2_O_3_	aurantiamide	alkaloid	[80]
72	412.68	412.70	C_29_H_48_O	beta-stigmasterol	steroids	[94]
73	414.69	414.70	C_29_H_50_O	sitosterol	steroids	[94]
74	424.49	424.5	C_25_H_28_O_6_	tetramethylcurcumin	diarylheptanoid	[93]
75	432.39	432.4	C_21_H_20_O_10_	tetrahydroxycurcumin	diarylheptanoid	[93]
76	536.89	536.9	C_40_H_56_	beta-carotene	miscellaneous	[86]
77	594.49	594.5	C_27_H_30_O_15_	kaempferol-3-rutinoside	flavonoid	[93]
78	610.49	610.50	C_27_H_30_O_16_	rutin	flavonoid	[82]
79	588.71	588.70	C_36_H_44_O_7_	terpecurcumin A	terpenoids	[95]
80	602.71	602.70	C_36_H_42_O_8_	terpecurcumin D	terpenoids	[95]

The metabolite profile results of curcuma sample performed through GC-MS and mass spectroscopy correspond with the data reported in the literature [1].

### 3.2. Screening and Classification of the Differential Metabolites

The 80 secondary metabolites identified through mass spectroscopy were assigned to multiple chemical classes: terpenoids and sesquiterpenoids (35%), amino acids (13.75%), diarylheptanoids (11.25%), flavonoids (10%), phenolic acids (8.75%), sterols and steroids (6.25%), fatty acids (6.25%), coumarins (1.25%), alkaloids (1.25%) and miscellaneous. Terpenoids and sesquiterpenoids, amino acids and diarylheptanoids represent 60% of all bioactive compounds from *Curcuma longa*. The distribution of identified phytoconstituients in various chemical categories is listed in Table 3.

**Figure 3:**
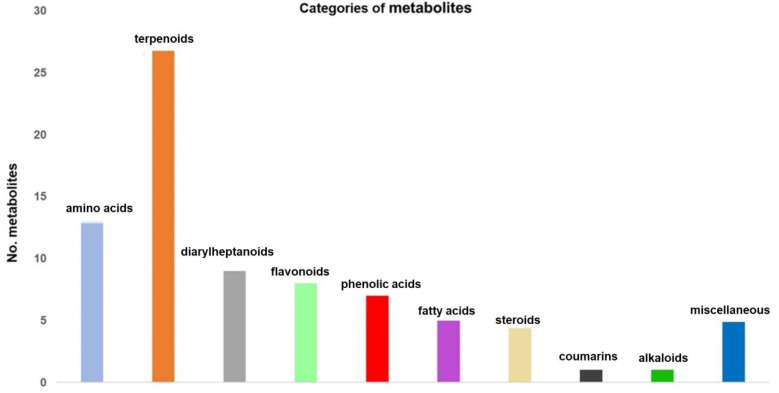
Metabolite classification bar chart of *Curcuma longa*.

Figure 3 presents the metabolite classification chart obtained on the basis of the data analysis reported in Table 3.

*Amino acids and peptides*: A total of 11 compounds were identified in the rhizome extract. The essential amino acids (valine, leucine, methionine, phenylalanine and arginine) represent about 45.45% of the total compounds. The non-essential amino acids (proline, aspartic acid, glutamic acid, cysteine, serine, glycine and alanine) represent a slightly higher proportion, accounting for about 54.55% of the total compounds [96,97,98,99,100]. About half of the amino acids identified in the curcuma rhizome sample (arginine, phenylalanine, glutamic acid, methionine, glycine, serine, aspartic acid and proline) act as antitumoral, neuroprotective, antiproliferative and immunomodulant agents [97,98,99,100,101].

*Terpenoids and sesquiterpenes* are the largest categories of phytoconstituents found in curcuma rhizome samples. Various studies have demonstrated their anti-inflammatory, antimicrobial, antioxidant, antiviral, antibacterial, antifungal, hepatoprotective, neuroprotective, analgesic and immune-modulation activities [74,89,96,97,98,99,100,101,102,103,104].

*Diarylheptanoids (curcuminoids*), another of the largest classes of bioactive compounds from curcuma, have been shown to exhibit anti-inflammatory, antioxidant, neuroprotective, antibacterial, antifungal, antitumor, hepatoprotective and antidiabetic activities [74,105,106,107].

*Flavonoids* are another class of metabolites identified in the curcuma sample. These bioactive phytoconstituents have remarkable pharmacological proprieties, including antioxidant, antitumoral, antimicrobial and anti-inflammatory activities, as well as effects against neurodegenerative diseases (Alzheimer’s disease) [74,96,101,108,109,110,111].

*Sterol and steroids* are secondary metabolites with high anti-inflammatory, antitumoral, antidiabetic, antioxidant, antiatherosclerotic, neuroprotective, immunomodulatory and cardiovascular protective activities [105,111,112].

Studies have reported that *fatty acids* exhibit anti-inflammatory, antioxidant, neuroprotective and cardiovascular protective activity [101,102,113].

*Coumarin* is a secondary metabolite with outstanding therapeutic proprieties, acting as a highly antitumoral and bacteriostatic agent [101,114,115].

*Phenolic acids* are reported to act as antioxidant, anti-inflammatory, antitumoral, neuroprotective and antimicrobial agents [82].

*Aurantiamides*, the alkaloids identified in the curcuma sample, exhibit antioxidant, analgesic, antiplatelet, anti-inflammatory and antitumor activity [116,117].

*Miscellaneous* compounds, for instance, 2-hydroxy-3-(hydroxymethyl)anthraquinone, found in the curcuma sample exert anti-inflammation, antitumoral and immunoregulation activity [118].

Studies have reported that beta-carotene exhibits highly antioxidant, anti-inflammatory, hepatoprotective and antitumoral activity [119].

### 3.3. Phytoengineered Carrier System

The development of a target-specific phytocarrier system imposes tailored assembled structures with pre-established morphostructural and biological properties able to exert complementary and synergistic therapeutic efficacy and trigger selective controlled delivery, release and activation in specific body regions, in addition to minimizing toxicity [120,121,122,123].

A nanocarrier system that collectively combines the specific features of silver nanoparticles (high biocompatibility, permeability and stability, with unique optical properties) with remarkable antioxidant, neuroprotective and anti-inflammatory effects of both components (curcuma and inorganic components) can increase the response to the specific physiological microenvironment and thus provide high efficiency through enhanced permeability and retention effects [8,14,15,16,17,22,23,24,25].

### 3.4. FT-IR Spectroscopy

**Figure 4:**
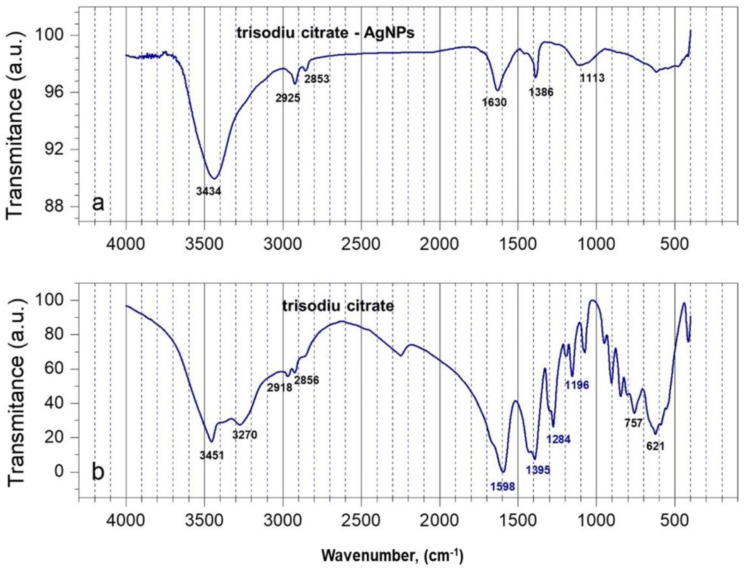
FTIR spectra of sodium-citrate-coated silver nanoparticles (**a**) and sodium citrate (**b**).

**Figure 5:**
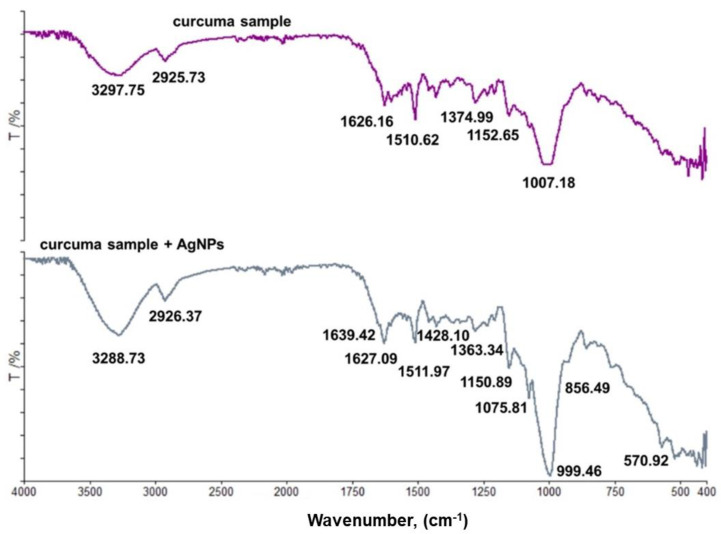
FT-IR spectrum of the curcuma sample and curcuma–AgNP carrier assembly.

The preparation of a phytocarrier system was investigated by FT-IR spectroscopy to identify the functional groups specific to its two components, curcuma and AgNPs. The individual FT-IR spectra of the curcuma, citrate-coated AgNPs and phytocarrier systems are shown in Figure 4 and Figure 5.

The FT-IR absorption bands identified in the curcuma sample are presented in Table 4.

The FTIR peak of AgNPs coated with trisodium citrate (surfactant) (Figure 4a) presents vibrational bands characteristic of a surfactant at 3434 cm^−1^ (associated with H-OH stretching vibration) and 2925 cm^−1^, at 2853 cm^−1^ (attributed to CH- asymmetric and symmetric stretching vibrations), 1630 cm^−1^ (associated with COO- stretching vibration) and 1386 cm^−1^ (assigned to C-H bending) [13].

The obtained results (Figure 5) confirm the successful development of a phytoengineered carrier delivery system. The obtained spectra of the carrier system include the characteristic peaks of curcuma at 3288 cm^−1^, attributed to the -OH group; at 2926.37 cm^−1^, assigned to C-H aromatic; at 1627 and 1511.97 cm^−1^ (C=C and diarylheptanoid heterocyclic ring, respectively); at 1150.89 and 1071.11 cm^−1^ (C–O and C–O–C bonds, respectively); at 882.44 and 816 cm^−1^ (C-O and CH vibration of aromatic rings), as well the AgNPs coated with surfactant (trisodium citrate) (Figure 4a,b) [135].

Moreover, the absorption bands at 1630, 1386, 1113 and 633 cm^−1^ that appear in synthesized AgNP solution (Figure 4b) are shifted to higher wavenumbers (1639.42, 1428.10, 1150.89 and 681 cm^−1^), suggesting the binding of AgNPs to the O–H, C=O, N-H and C–O functional groups of curcuma phytoconstituents (Figure 5 and Table 4) [21,22,23,24,25,126,128,129,136].

Furthermore, in the IR spectra of the new phytocarrier system, several discernible changes occur compared to the spectrum of curcuma, in particular, in the case of the hydroxyl vibrations (O-H stretching, H-bonded alcohols and phenol groups), which are shifted to lower wavenumbers, indicating that this functional group is involved in the binding of AgNPs [19,21,22,23,24,25,127,137,138,139,140].

### 3.5. UV-Vis Spectroscopy

This technique was used to obtain insight into the newly developed carrier system. UV-VIS analysis provides information on the characteristics of AgNPs.

Various studies have reported that the optical properties of AgNPs are influenced by particle size, degree of dispersion, coating and chemical stability [11,13,19,25,141,142].

**Figure 6:**
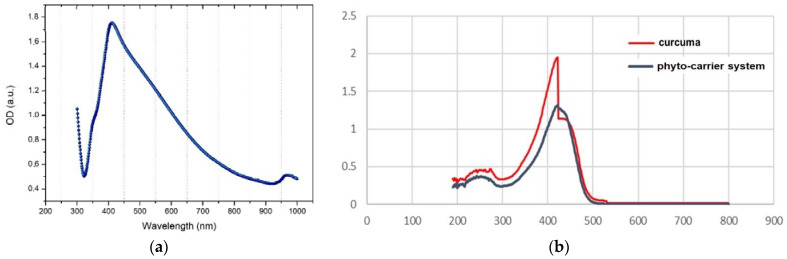
(**a**) UV-Vis spectra of citrate-coated AgNPs; (**b**) UV-Vis spectra of the phytocarrier system and curcuma sample.

As shown in Figure 6a, AgNPs present a plasmonic band at 417 nm, which can be assigned to citrate-coated AgNPs, as well as another two weak absorbance peaks at 368 and 970 nm [143,144].

The sharp absorption maximum (λmax) at 417 nm (Figure 6a) indicates that the size of the synthesized AgNPs is in the 20–120 nm range and dispersed [13,145]. Additionally, a single surface plasmonic peak (SPP) in the UV-Vis spectra suggests that the AgNPs are mostly spherical [145,146].

The shape of the curcuma absorption spectrum (Figure 6b) showed a maximum absorption band at a wavelength of 424.5 nm (attributed to curcuminoids) and a shoulder near 360 and 464 nm (assigned to bet-carotene), as well as a weak absorption band at 261 nm (attributed to diarylheptanoids and terpenocurcumins) [95,130,146,147,148].

A comparison of the shape of the spectrum of curcuma and that of the new phytocarrier system reveals notable differences (Figure 6b). The surface plasmon band of the plasmon of the new phytocarrier system was detected at 415 nm, with a weak peak at 262 nm from curcuma. These results confirm the achievement of the phytocarrier system.

### 3.6. X-ray Diffraction Spectroscopy

The XRD analysis was used to investigate the crystalline structure of the carrier system and its components.

**Figure 7:**
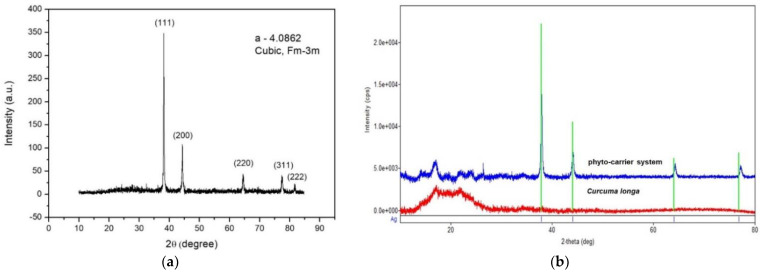
(**a**) Powder XRD patterns of AgNPs; (**b**) XRD patterns of curcuma and the phytocarrier system.

Figure 7a,b display the XRD patterns of the AgNPs, curcuma sample and the new phytocarrier system.

The specific XRD spectrum of AgNPs (Figure 7a) exhibits a cubic face-centered crystal lattice amd a space group Fm-3m crystalline structure. The XRD peaks at 2θ values of 38.5°, 46.5°, 64.4° and 78.5° can be assigned to the (111), (200), (220) and (311) lattice planes, respectively [139,149].

The mean diameter (D) of the silver crystallites calculated using the Debye–Scherrer formula is about 120 nm.

The XRD pattern of curcuma (Figure 7b) is in the range of 14.8–22.4°, with large bands and weak peaks characteristic of amorphous phases that can be attributed to curcuma constituents, namely dietary fibers and minerals, such as hydroxides [1,127]

There are several notable differences in the XRD pattern for the new carrier system, dispite similarities to that of curcuma. The characteristic bands of the plant in the region between 15.8 and 22.3 are considerable attenuated. Additionally, AgNP peaks can be easily observed at 38.2, 44.1, 64.4 and 78.5°. The estimated average diameter of the AgNPs from the phytocarrier system is ~30 nm, consistent with the SEM results.

### 3.7. Scanning Electron Microscopy (SEM)

**Figure 8:**
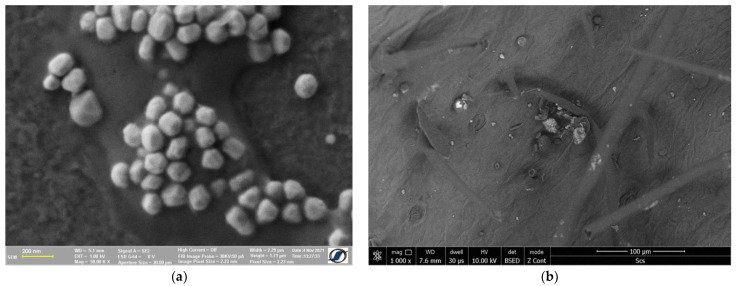
(**a**) Two-dimensional image of the AgNPs (citrate coated) obtained by the SEM technique. (**b**) Two-dimensional image of the curcuma sample obtained by the SEM technique (magnitude: 100 μm).

Based on the SEM micrograph of AgNPs (Figure 8a), the metal particles were well-dispersed with a smooth and spherical shape of *ca.* ~100 nm [13,150,151,152,153].

**Figure 9:**
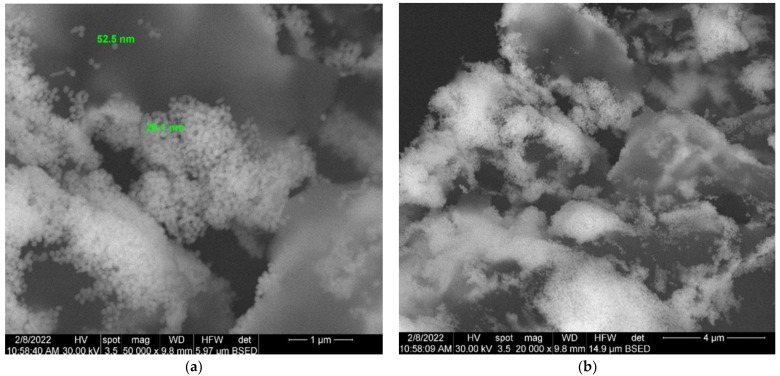
(**a**) Two-dimensional image of the phytocarrier system obtained by the SEM technique (magnitude: 1 μm). (**b**) Two-dimensional image of the phytocarrier system obtained by the SEM technique (magnitude: 4 μm).

The morphology of the curcuma sample (Figure 9b) displays a surface with a fibrous structure, with irregular, porous areas measuring approximately 6 µm. The presence of pores suggests easy fixation of metal nanoparticles on the surface of the plant sample.

The morphology of the phytocarrier system indicates the presence of metallic nanoparticles and agglomerations of spherical AgNPs with dimensions between 20 and 53 nm, both on the surface of the plant particles and loaded into the pores of herb particles (Figure 9a,b) [127].

The size of these AgNPs within the phytocarrier system (Figure 9a) observed under SEM does not significantly differ from those measured using XRD (Figure 7b) and according to UV-Vis spectroscopy (Figure 6b) [154].

However, a considerable variation can be observed between the synthesized AgNPs (about 100 nm) and those from the phytocarrier system (20–53 nm). The significant reduction in the size of AgNPs observed in the phytocarrier system compared to the size of the synthesized AgNPs can be attributed to the specific experimental conditions under which the phytocarrier system was obtained, confirming the variation in the size of the nanoparticles depending on the experimental parameters reported in the literature [155]. On the other hand, it can be assumed that as a result of filtration, the larger silver nanoparticles were better retained at the liquid/filter interface, as larger particles are more prone to form aggregates in the pore region. Additionally, plant pore size was an exclusion parameter for larger silver nanoparticles, allowing only nanoparticles below a certain diameter to enter the pores and be transffered into the herb.

### 3.8. Dynamic Light Scattering (DLS)

DLS analysis was used to obtain information about the particle size and particle size distribution both in the case of citrate-coated AgNPs and for the phytocarrier system.

Ultrapire water was used because the DLS principle is based on the Brownian motion of dispersed particles in a liquid. The intensity of light scattering varies depending on the dispersion of nanoparticles of varying sizes.

**Figure 10:**
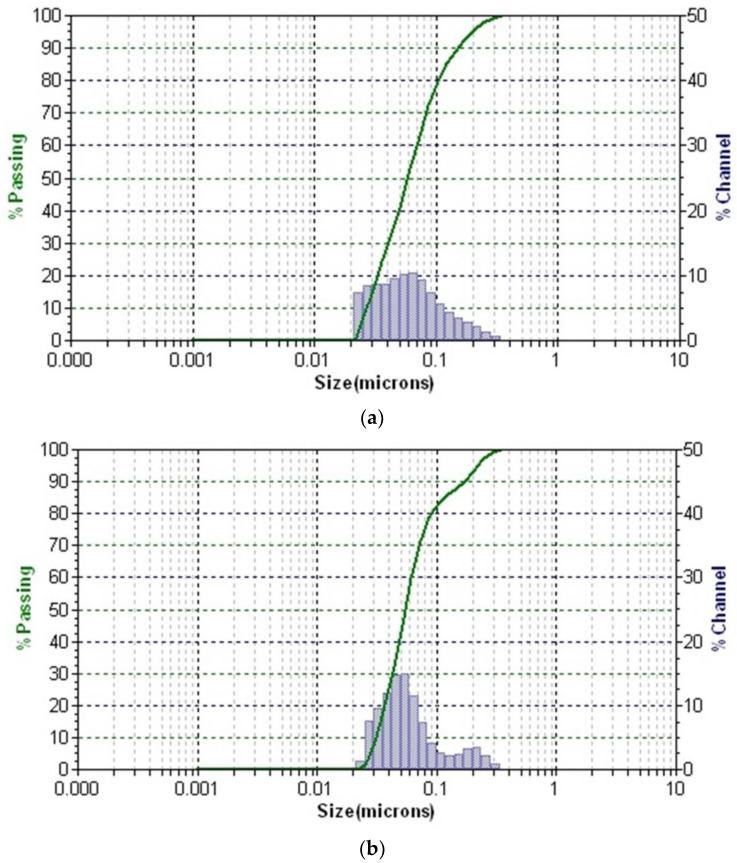
(**a**) DLS pattern of citrate-coated AgNPs. (**b**) DLS pattern of the phytocarrier system.

Figure 10a shows the average mean particle size of citrate-coated AgNPs.

The particles have a size of 100 nm, corroborating the UV-Vis and SEM results.

Figure 10b presents the DLS analysis of the phytocarrier system.

The DLS curve of the phytocarrier system displays two peaks that can be attributed to AgNPs and curcuma nanoparticles.

**Figure 11:**
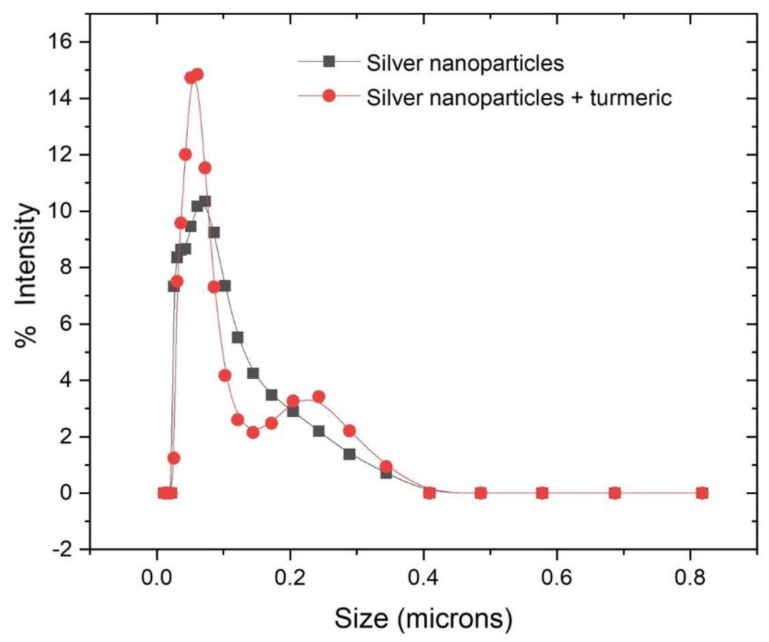
DLS curve of AgNPs and the phytocarrier system.

Superimposition of the individual AgNPs and the phytocarrier system DLS patterns (Figure 11) reveals the presence of peaks corresponding to two types of nanoparticles with varying sizes.

The results of the DLS analysis for the phytocarrier system (Figure 10b and Figure 11) suggest that the particle sizes are distributed in a narrow range. The mean diameter of AgNPs from the phytocarrier system is 52 nm. The second mean of the hydrodynamic diameter, attributed to curcuma nanoparticles, is about 220 nm. Because the mean size provided by the DLS analysis refers to the hydrodynamic diameter, the measured nanoparticles dimensions are often seem larger than those obtained by SEM analysis. The AgNPs tend to form aggregates with settled fractions visible from the first day [156,157]. However, this phenomenon was not observed in the case of the phytocarrier system, probably due to the low density of the curcuma.

In addition, well-dispersed particles (AgNPs and curcuma) from the phytocarrier system were identified (Figure 11). These results suggest high stability of the phytocarrier system without absorbance spectrum modifications [158].

### 3.9. UHR-TEM

**Figure 12:**
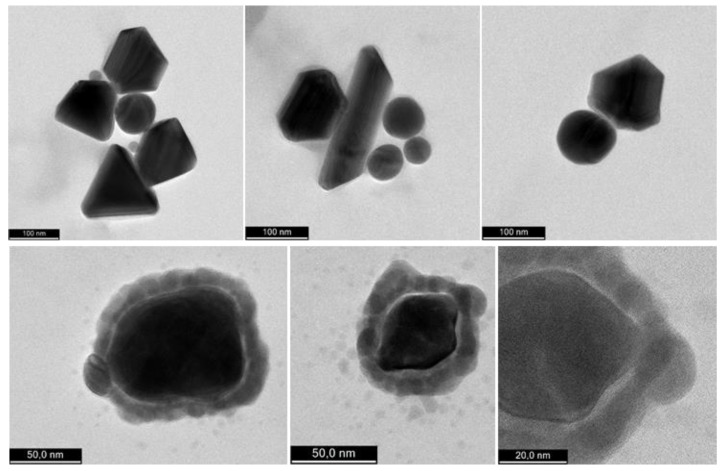
TEM images of AgNPs (**top row**) and the phytocarrier system (**bottom row**).

TEM analysis provided details about the size and shape of the silver nanoparticles and phytocarrier complex. As shown in Figure 12, the unfiltered AgNPs were polymorphic, with spherical, triangular, prismatic and rod-like shapes and sizes distributed in the range of approximately 35 nm to 150 nm, confirming the SEM analysis. Furthermore, given that the SEM image of curcuma appeared to be less detailed, we decided to double-filter the curcuma suspension for the UHR-TEM measurements, which allowed only the finest curcuma nanoparticles to pass through. As a result, an approximately 15 nm thick corona of curcuma nanoparticles was formed on the AgNP surface (Figure 12), indicating an increased attraction between the organic and inorganic nanostructures mediated mostly by physical forces.

The curcuma corona appears to be composed of individual nanoparticles of approximately 10–15 nm in diameter.

### 3.10. Zeta Potential

The zeta potential of the nanoparticles was measured in order to evaluate the surface charge potential, which is an important parameter for evaluation of suspension stability [159].

The zeta potential value of AgNPs was −34.49 mV, indicating the high stability of the suspension. A negative value was expected, as the nanoparticles were stabilized with sodium citrate and ascorbic acid. The zeta potential changed to −22.34 mV for the phytocarrier system, indicating the stability of the solution. This negative surface charge indicates the high biocompatibility of the obtained phytocarrier system [160].

### 3.11. EDS Spectroscopy

**Figure 13:**
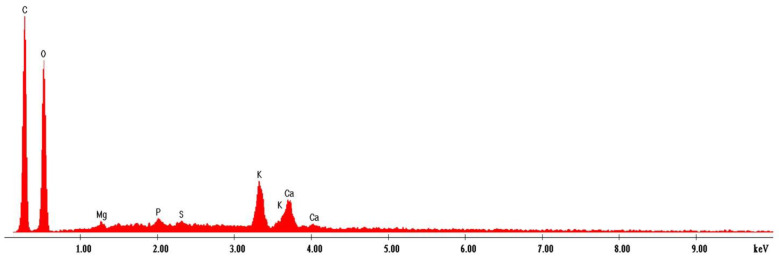
EDX spectrum of curcuma sample.

**Figure 14:**
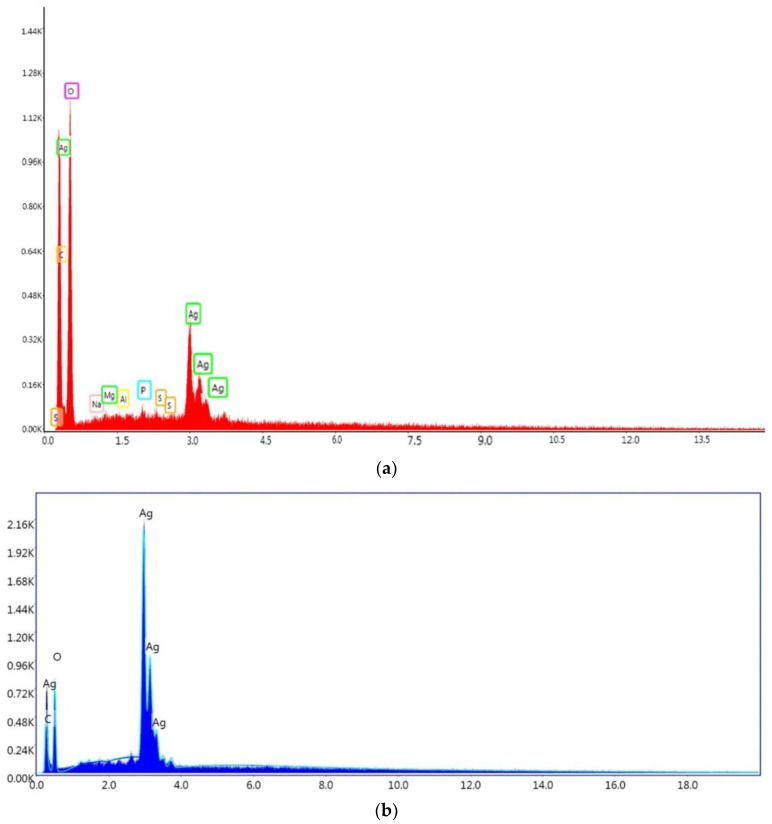
(**a**) EDX spectrum of phytocarrier system. (**b**) EDX spectrum of phytocarrier system (O K/C K/AgL phase).

The EDS spectra of the phytocarrier system and curcuma sample are shown in Figure 13 and Figure 14, respectively.

The energy-dispersive X-ray (EDX) spectrum results of the phytocarrier system revealed strong signals in the silver region, confirming the presence of silver in the analyzed micro area (Figure 14a,b).

**Figure 15:**
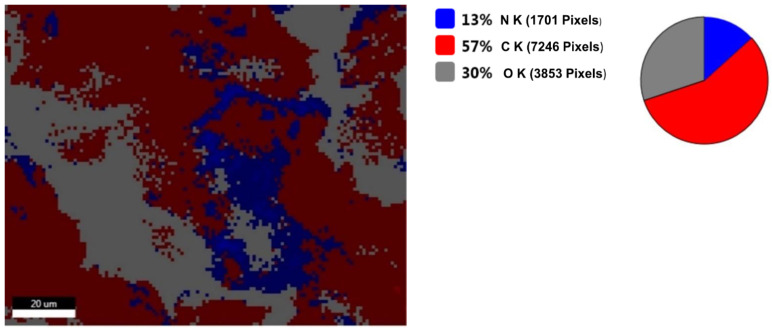
*Curcuma longa* SEM live map.

Figure 15 displays the live map for curcuma and the distribution of the identified elements.

**Figure 16:**
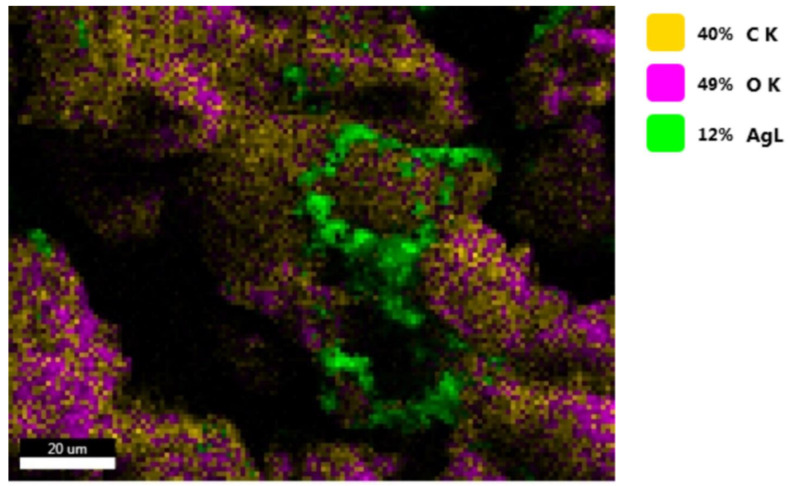
SEM live map of the new phytocarrier system.

The comparative analysis presented in Figure 16 showing a live map for herb and phytocarrier system (Figure 16) highlights the presence of differences in terms of the proportion of identification elements in the samples resulting from the formation of the new phytocarrier system.

The comparative analysis presented in Figure 15 showing live map for curcuma and the phytocarrier system (Figure 16) highlights the presence of differences in terms of the proportion of identification elements in the two samples resulting from the formation of phytocarrier system.

The results of SEM, EDX and SEM live maps confirm the achievement of the new phytocarrier system.

### 3.12. Screening of Antioxidant Activity

The total antioxidant capacity (TAC) of a particular plant results from the collective action of the categories of antioxidants of which it is composed [52].

Generally, in vitro and in vivo methods are used to estimate the total antioxidant capacity of natural compounds.

In vitro techniques are classified as noncompetitive (based on an electron transfer (ET) mechanism) or competitive measurements (based on hydrogen transfer (HAT)) [45,46,52,53].

Despite the availability of a standardized method, the most popular techniques are non-competitive, non-enzymatic assays (Folin–Ciocalteu reaction, DPPH, ABTS/TEAC, CUPRAC, ABTS and FRAP) for in vitro determination of antioxidant capacity in the presence of a chromogenic agent [45,46,52,53].

The antioxidant activity of the phytocarrier system is a result of the complementary and synergistic action of each component (curcuma and AgNPs). More than 80 secondary metabolites from 10 categories were identified in the curcuma sample. Therefore, to reflect the antioxidant properties of the new phytocarrier system more accurately, we used three in vitro, non-competitive, non-enzymatic, distinctive assays (DPPH, Folin-Ciocalteu and cyclic voltammetry).

### 3.13. DPPH (1,1-Diphenyl-2-Picrylhydrazyl) Free Radical Scavenging Assay

DPPH is a simple, fast assay widely used to determine the total antioxidant capacity of a liquid or solid sample. It is based on reduction of DPPH, a stable free radical, through a single electron transfer (ET)-type mechanism [45,46,52,53].

Thus, the antioxidant capacities of the new phytocarrier system and curcumin were evaluated and compared to three antioxidant standards (β-carotene, ascorbic acid and curcumin). These standards were selected based on the chemical screening results, which highlighted the presence of beta-carotene and curcumin in the curcuma sample. Moreover, studies have reported a high content of ascorbic acid in curcuma [161].

**Figure 17:**
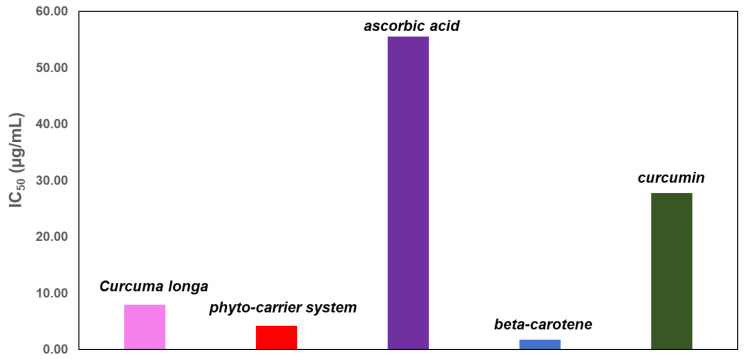
Graphic representation of DPPH results expressed as IC_50_ (µg/mL).

The obtained results are presented in Table 5 and Figure 17.

**Table 5:** IC_50_ values for curcuma, the phytocarrier system and standard antioxidants (ascorbic acid, curcumin and beta-carotene).

Sample	Curcuma	Phytocarrier System	β-Carotene	Curcumin	Ascorbic Acid
**IC_50_ (µg/mL)**	7.98 ± 0.12	4.19 ± 0.06	2.16 ± 0.13	45.46 ± 0.11	55.57 ± 0.18

Based on the obtained IC_50_ values and given that lower IC_50_ values correspond to higher antioxidant activity, the new phytocarrier system has higher antioxidant activity than that of curcuma, ascorbic acid and curcumin but lower than antioxidant activity than that of the beta-carotene standard.

Research has shown that the polarity of solvents significantly influences not only the extraction yield but also their capacity for antioxidant activity [81].

Therefore, only the beta-carotene solvent differed from that of the other samples (curcuma, curcumin, ascorbic acid and the phytocarrier system). Thus, a mixture of solvents with varying polarities (methanol-dichloromethane) was used, owing to its poor solubility in methanol, which can explain the obtained IC_50_ value of beta-carotene.

### 3.14. Folin–Ciocalteu Assay

The Folin–Ciocalteu assay measures the reducing capacity of an antioxidant based on electron transfer and is considered one of the most accessible and facile methods for evaluation of the total phenolic content (TPC) of a particular substrate [46,54,162,163].

The total polyphenolic content (TPC) of the phytocarrier system and the curcuma sample was determined; the obtained results are presented in Table 6.

According to the obtained data, the total polyphenolic content detected in the case of the new phytocarrier system is more than 50% higher than that of the curcuma sample.

The higher antioxidant capacity of the phytocarrier system compared to the curcuma sample can be attributed to the synergistic action of AgNPs, with a catalytic role played by curcuma polyphenolic compounds [164,165].

### 3.15. Cyclic Voltammetry

The cyclic voltammetry method provides electrochemical measurements of antioxidant characteristics using a redox-based mechanism to determine total antioxidant capacity. This method is reliable, fast, simple and cost-efficient, providing affordable and accurate information on the reducing capacity of active components [52,54,166,167]

A cyclic voltammogram provides data on the total antioxidant capacity of a substrate without providing information on the individual contribution of specific antioxidant categories. The total antioxidant capacity is the result of the combined action of two key parameters: the oxidation potential (which reflects the reducing power of a particular compound) and the anodic peak current intensity (Ia) (corresponding to the concentration of the bioactive component). Low oxidation potential values indicate the ability of biomolecules to donate electrons, as well as implicit high antioxidant activity [167,168,169].

The cyclic voltammetric (Trolox equivalent antioxidant capacity (TEAC) electrochemical assay) method was performed to determine the total antioxidant capacity (TAC) of the phytocarrier system and its components (curcuma and AgNPs). This method investigates the ABTS radical (2,20-azinobis(3-ethylbenzothiazoline-6-sulphonic acid)) and establishes the catalytic voltammetric currents.

The Trolox equivalent antioxidant capacity (TEAC) assay is based on the ability of a given compound with antioxidant properties to scavenge ABTS∙^+^ radicals or ABTS^2+^ cations [58,170,171].

**Scheme 1:**
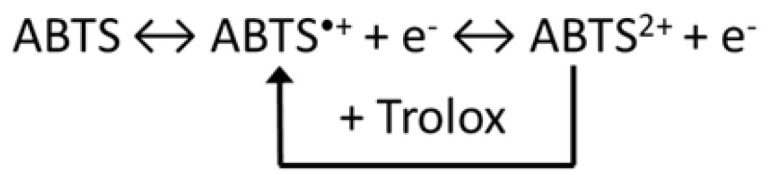
Redox chemistry of the ABTS/Trolox system.

In the present study, we exploited the ability of Trolox to reduce ABTS^2+^ cations according to the following scheme (Scheme 1) [58,171].

**Figure 18:**
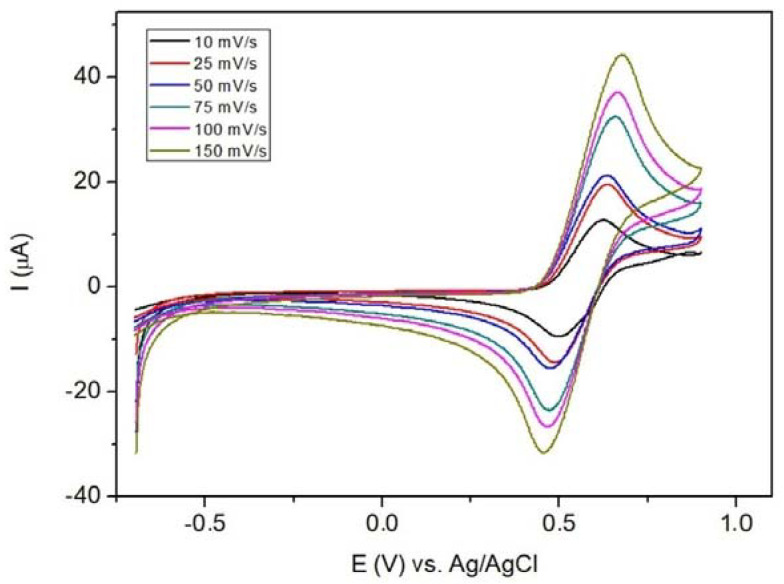
CVs in 0.12 mM ABTS∙^+^ in a 1:1 water/methanol mixture with 0.05 M KCl recorded at several scan rates.

The stability of the ABTS∙^+^ radical was monitored through CV measurements at several scan rates (Figure 18).

The absence of redox peaks at 230/231 mV corresponding to the discharge of the ABTS/ABTS^+^ pair suggests that the ABTS working solutions contained only ABTS^+^ species, as confirmed by the appearance of characteristic redox peaks at 489/490 mV.

**Figure 19:**
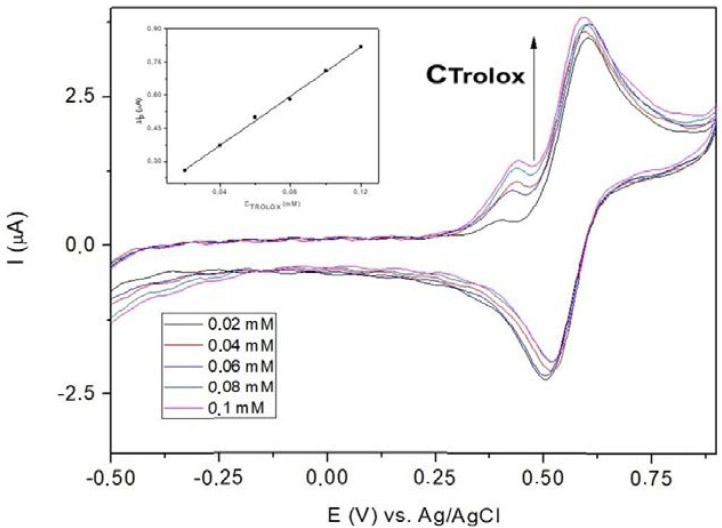
Changes in CVs features of ABTS∙^+^ solution in the presence of increased concentrations of Trolox (scan rate 50 mV/s). Inset: calibration plot for the TEAC electrochemical assay obtained from the CVs of ABTS∙^+^ in the presence of increasing concentration of Trolox.

In the presence of various amounts of Trolox, the CVs of ABTS underwent significant modifications. For Trolox concentrations exceeding 0.02 M, the anodic peak increases linearly with the amount of Trolox, whereas the backward catodic peak decreases correspondingly (Figure 19).

According to Scheme 1, when Trolox is added to the cell, it reacts with the ABTS^2+^ cation, the product of electrode oxidation of ABTS∙^+^. Thus, the electrochemical species, ABTS∙^+^, is regenerated and can be reoxidized on the electrode surface. This phenomenon leads to an increase in the anodic current, accompanied by a simultaneous decrease in the cathodic current. Higher concentrations ultimately yield higher oxidation peaks, as more ABTS∙^+^ species are regenerated during the voltammetric assay.

ΔI_p_ is defined as the difference between the measured oxidation peak currents in the presence of corresponding Trolox (I_p,x_) and in the absence of Trolox (I_p,0_).

Thus, the regression plot of ΔI_p_ vs. Trolox concentration provides the following equation (Equation (1)):

ΔI_p_(μA) = (5.571 ± 0.13)∙C_Trolox_ (mM) + (0.15 ± 0.01) (with R^2^ = 0.99705) (1)

This plot was used to estimate the antioxidant capacity (TEAC) of curcuma, AgNPs and the phytocarrier system.

**Figure 20:**
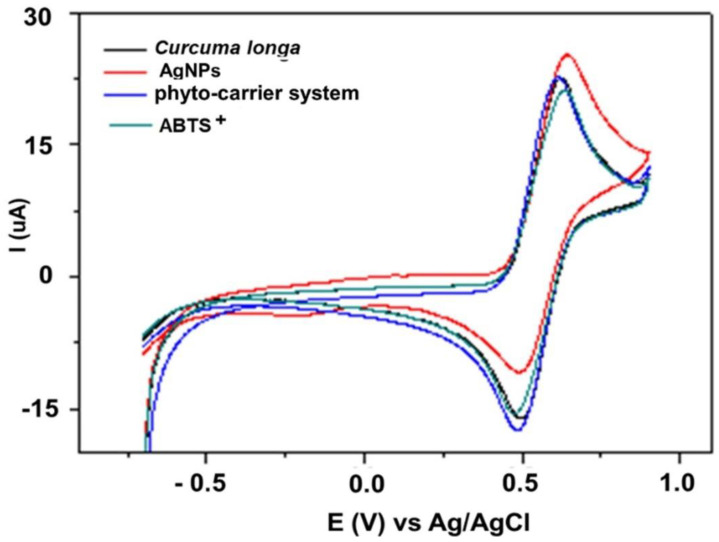
Increase in anodic peak currents in ABTS∙^+^ voltammetric measurements in the presence of curcuma, AgNPs and the phytocarrier system (5% volummetric ratio) (scan rate: 50 mV/s).

The optimized volumetric ratio provided the appropriate peak currents for interpolation in the calibration plot. An increase in the oxidation current of ABTS∙^+^ was observed, as in the presence of Trolox (Figure 20).

The electrochemical TEAC assay provided the following results (expressed as mmole/L Trolox): 6.6 ± 0.4, 3.7 ± 0.2 and si 4.2 ± 0.25 (n = 10) for the phytocarrier system, citrate-coated AgNPs and curcuma, respectively.

The value was larger than that suggested by the TEAC data (spectrophotometric method) reported in literature for curcuma extract (TEAC_curucma_ = 0.00292 mmol/L) [172,173].

However, the experimental conditions differed, making the comparison of the results of various studies unrealistic [52].

The TEAC values indicate that the phytocarrier system has a higher antioxidant activity than that of each component (curcuma and AgNPs).

This high antioxidant activity of the phytocarrier system can be attributed to the synergistic and complementary action of the antioxidants in curcuma and the antioxidant mechanism of AgNPs (quenched free radicals by donating or accepting electrons) in the presence of the various bioactive compounds in curcuma [165,174].

In addition, the structural characteristics of AgNPs (shape and size) influence the antioxidant activity [175].

Various studies have reported that AgNPs influence the antioxidant activity, triggering ROS production through a reduction in reactive oxygen species [175,176,177].

Furthermore, the antioxidant activity of citrate-coated AgNPs is the result the antioxidant mechanism of the metal nanoparticles, as well as sodium citrate and ascorbic acid from the coating of the nanoparticles [178,179,180].

## 4. Conclusions

In this study, a new phytocarrier system with unique optical properties and high antioxidant activity was developed. The metabolite profile of *Curcuma longa* was determined. The biological activities were discussed for each metabolite category. The achievement of the curcuma–citrate-coated AgNP carrier system was confirmed through UV-Vis FTIR, EDX, XRD, DLS, zeta potential, SEM and TEM studies. The size distribution of AgNPs and curcuma particles was measured through DSL analysis. The stability of AgNPs and the phytocarrier system in aqueous suspensions was determined using zeta potential analysis.

A combination of methods (DPPH, Folin-Ciocalteu and cyclic voltammetry) was used to evaluate the antioxidant properties of the proposed phytocarrier system. The results show a significantly higher antioxidant activity of the phytocarrier than that of curcuma or AgNPs. However, further research is necessary to investigate the biological activity and biocompatibility of the new phytocarrier system. This study motivates future therapeutic research in the fields of innovative neurodegenerative agents.

## Figures and Tables

**Table 3:** Classification of bioactive phytoconstituents from the *Curcuma longa* sample organized according to chemical category.

Chemical Class	Metabolite Name
**Flavonoids**	dehydrozingerone
	apigenin
	naringenin
	epicatechin
	quercetin
	myricetin
	kaempferol-3-rutinoside
	rutin
**Terpenoids**	cymene
	limonene
	terpinene
	tymol
	thujone
	terpineol
	eugenol
	zingiberone
	β-curcumene
	zingiberene
	turmerone
	germacrone
	turmerol
	zingibrene
	curzerene
	α-costal
	santanol
	α-copaen-11-ol
	α-bisabolol
	curzerenone
	glechomanolide
	curcumenol
	curdione
	zederone
	procurcumadiol
	bisacurone
	turmerol
	terpecurcumin A
	terpecurcumin D
**Diarylheptanoids**	bisdemethoxycurcumin
	1,7-bis(4-hydroxyphenyl)-3,5-heptanediol
	1,5-bis(4-hydroxy-3-methoxyphenyl)-1,4-pentadien-3-one
	curcumin
	cyclocurcumin
	1,2-dihydrocurcumin
	dimethoxycurcumin
	tetramethylcurcumin
	tetrahydroxycurcumin
**Amino acids**	glycine
	alanine
	serine
	proline
	valine
	leucine
	aspartic acid
	glutamic acid
	methionine
	phenylalanine
	arginine
**Phenolic acids**	cinnamic acid
	protocatechuic acid
	p-coumaric acid
	gallic acid
	caffeic acid
	ferulic acid
	chlorogenic acid
**Sterols**	gitoxigenin
	cholesterol
	campesterol
	beta-stigmasterol
	sitosterol
**Fatty acids**	palmitic acid
	linoleic acid
	oleic acid
	stearic acid
	methyl stearidonate
**Coumarins**	coumarin
**Alkaloids**	aurantiamide
**Miscellaneous**	trimethylbenzene
	undecan-2-one
	dodec-11-en-2-one
	2-hydroxy-3-(hydroxymethyl)anthraquinone
	beta-carotene

**Table 4:** The characteristic absorption bands attributed to different metabolites identified in *Curcuma longa*.

Metabolites	Wavenumber (cm^−1^)	Ref.
Amino acids	3400; 3330–3130; 2530–2760; 2130; 1724–1754 1687, 1675, 1663, 1652, 1644, 1632, 1621, 1611, 1610–1660, 1500–1600	[124]
Flavonoids	4000–3125, 3140–3000, 1670–1620, 1650–1600, 1600–1500, 1450–1490	[125,126]
Terpenoids	2939, 1740, 1651, 810	[127]
Phenolic acids	1800–1650, 1734, 1720, 1627, 1522, 1440, 1410, 1420–1300, 1367, 1315, 1255, 1170–1100	[127]
Fatty acids	3020–3010, 2924–2915, 2855–2847, 2800-2900, 1746, 1710, 1250, 720	[128,129]
Dyarylheptanoids	2100-2550, 1620-1740	[130]
Coumarin	600–900, 1028, 1254, 1450, 1489, 1608, 1715, 2963, 3061, 3381	[131]
Beta-carotene	965, 1385, 1635, 2920	[132]
Alkaloid	720, 755, 1570, 1639, 1650, 3400	[133]
Sterols	740.5, 1063, 1192, 1383, 1466, 2937, 3427	[134]

**Table 6:** Total polyphenolic content in curcuma and the phytocarrier system.

Sample	Total Phenolic Content (µg/mL)
phytocarrier system	127.47 ± 0.03
*Curcuma longa*	76.02 ± 0.06

## Data Availability

All data are contained within the article.
